# Editorial: Genetic insights and diagnostic innovations in cerebrovascular and cerebrospinal fluid disorders

**DOI:** 10.3389/fneur.2025.1691759

**Published:** 2025-10-21

**Authors:** Ling Li, Haipeng Liu, Linfang Lan, Hao Yu

**Affiliations:** ^1^Department of Neurology, Zhoushan Hospital, Wenzhou Medical University, Zhoushan, Zhejiang, China; ^2^Centre for Intelligent Healthcare, Coventry University, Coventry, United Kingdom; ^3^National Medical Research Association, Leicester, United Kingdom; ^4^Department of Neurology, The First Affiliated Hospital of Sun Yat-sen University, Guangzhou, China; ^5^Department of Medical Genetics and Center for Rare Diseases, Second Affiliated Hospital, Zhejiang University School of Medicine and Zhejiang Key Laboratory of Rare Diseases for Precision Medicine and Clinical Translation, Hangzhou, Zhejiang, China

**Keywords:** cerebrovascular diseases (CVDs), neuroimaging, multi-omics data analysis, molecular genetics, artificial intelligence

Genetics-centered precision neurology is reshaping both research and clinical practice in cerebrovascular disease and cerebrospinal fluid-related disorders. Technological advancements in multimodal neuroimaging, standardized laboratory testing, next-generation sequencing, transcriptomics, and extracellular-vesicle analytics are collectively accelerating the acquisition, management, processing, and interpretation of novel biomarkers for clinical practice. The impact is evident in earlier etiologic clarification, finer-grained risk stratification, and biomarker-informed monitoring that spans the acute phase through rehabilitation. Within this Research Topic, Genetic Insights and Diagnostic Innovations in Cerebrovascular and Cerebrospinal Fluid Disorders, we assembled 13 contributions—reviews, bioinformatics analyses, clinical cohort studies, neuroimaging investigations, and preclinical experimentation—that collectively illustrate how genetics and novel biomarkers are being applied to cerebrovascular and cerebrospinal fluid-related diseases. Taken together, these studies provide up-to-date examples of how molecular insights can be translated into implementable diagnostic tools and mechanism-informed therapeutic strategies. The details are summarized in Supplementary Table 1.

We observed that multiple low-cost and readily obtainable blood biomarkers show promise for risk stratification and outcome prediction. Building on a randomized study, Mitra et al. showed that Short Message Service–guided exercise improved post-stroke six-minute walk test (6MWT) performance and attenuated the decline in brain-derived neurotrophic factor (BDNF). Changes in choline acetyltransferase (ChAT) activity and in the ChAT/butyrylcholinesterase (ChAT/BChE) index correlated with the 6MWT outcomes, with stronger signals observed in women for ChAT activity. This synchronized acquisition of biomarker and behavioral endpoints highlights the potential of peripheral markers as tools for monitoring treatment response.

In an observational analysis of 1,470 older adults with acute ischemic stroke (AIS), Huang et al. identified a non-linear inverse association between a lower hemoglobin-to-red blood cell distribution width ratio (HRR) and a higher risk of unfavorable 3-month outcomes. Restricted cubic spline modeling revealed an optimal inflection point at 10.70, with an area under the curve of approximately 0.64. Lower HRR values signaled greater risk. As a zero-additional-cost index derived from routine hematology tests, the HRR may offer practical value for bedside risk stratification.

In a cohort of AIS patients treated with intravenous thrombolysis, Li et al. evaluated the etiology-dependent prognostic value of lymphocyte-related ratios. The neutrophil-to-lymphocyte ratio (NLR) consistently predicted 90-day outcomes across the Trial of Org 10,172 in Acute Stroke Treatment (TOAST) subtypes with subtype-specific cutoffs and was associated with adverse outcomes in each category, whereas the lymphocyte-to-monocyte ratio (LMR) showed predictive value primarily in the large-artery atherosclerosis subtype.

Using a large United States adult cohort and multivariable logistic regression, Ye et al. examined the neutrophil percentage-to-albumin ratio (NPAR) in relation to stroke prevalence and found that a higher NPAR was associated with greater prevalence. These findings provide new insights for primary prevention and support the NPAR as a practical tool for estimating stroke likelihood.

Drawing on a clinical database of critically ill patients with acute brain injury, Wang J. et al. assessed the stress hyperglycemia ratio (SHR) and showed that it independently predicts both short- and long-term mortality. When combined with the Glasgow Coma Scale (GCS) and ventilation status, the SHR further improved risk stratification, supporting its use as a practical and feasible quantitative metric in intensive care settings.

In a large cross-sectional analysis of the National Health and Nutrition Examination Survey (NHANES), Xu H. et al. used weighted multivariable models with stratified interaction testing and provided the first population-level evidence of an independent inverse association between the serum klotho and stroke risk. The association was consistent across most subgroups. These results suggest that anti-aging endocrine pathways may modulate cerebrovascular risk, indicating the possibility of developing hormone biomarker panels for risk stratification.

With respect to genetics and large-vessel structural phenotypes, Wang J.-W. et al. examined the association between the apolipoprotein E (APOE) genotype and extracranial carotid artery (ECA) tortuosity in a Chinese cohort and found that the ε2 allele may be associated with the increased tortuosity of the ECA, whereas the ε4 allele might be a protective factor. These observations suggest that lipid-metabolism genotypes may influence the geometry of cerebral arteries.

By integrating peripheral blood transcriptomes from multiple Gene Expression Omnibus cohorts, Wang and Liu further identified ABCA1, CLEC4E, and IRS2 as potential key biomarkers and therapeutic targets for cardioembolic stroke and ischemic stroke, serving as shared feature genes. Their expression correlates closely with neutrophil infiltration and autophagy activation, and a nomogram based on these markers demonstrates potential clinical applicability. This progression from single-gene signals to systems-level networks suggests translatable diagnostic and therapeutic targets.

In symptomatic intracranial atherosclerotic stenosis (sICAS), Xu X. et al. linked cerebral perfusion patterns to infarct topography and early neurological outcomes. Specific perfusion abnormality profiles were associated with cortical and subcortical infarct distributions, as well as short-term clinical trajectories, underscoring the central role of hemodynamic compromise in the risk stratification of sICAS and identifying the candidates for intensified hemodynamic management. The penumbra-infarct core mismatch volume in CT perfusion, with a Tmax of >4s defining the penumbra, was associated with early neurological outcomes in patients with sICAS.

On the translational front, using a murine transient middle cerebral artery occlusion model, Wang L.-P. et al. evaluated the anti-inflammatory and blood–brain barrier (BBB)–protective effects of oligodendrocyte precursor cell (OPC) transplantation. OPCs reduced neuroinflammation, preserved BBB integrity, decreased infarct volume, and improved neurobehavioral outcomes, with benefits associated with Wnt/β-catenin signaling. These findings indicate a promising therapeutic strategy for ischemic stroke.

From a phytochemistry perspective, Yu et al. reviewed natural compounds derived from traditional Chinese medicine (TCM) that regulate microglial polarization to achieve neuroprotection after ischemic stroke. Multiple classes of compounds inhibit pro-inflammatory polarization and/or promote protective polarization, thereby exerting neuroprotective effects within a multitarget network. The review systematically catalogs candidate molecules and pathways, summarizes delivery innovations, and emphasizes the need for standardized pharmacology, pharmacokinetics, and quality control to advance standardized and personalized TCM treatment and management of ischemic stroke.

Liang et al. presented a comprehensive synthesis of blood-based biomarkers in ischemic stroke, covering coagulation and fibrinolysis pathways, endothelial dysfunction markers, inflammatory mediators, neuronal and axonal injury markers, and extracellular vesicles with their circular RNAs. The review also surveys contemporary detection platforms and assay methodologies, providing critical guidance for clinical implementation. Across these categories, many candidates show promise for etiologic subtyping, early neurological deterioration, and prognostic assessment, thereby bridging molecular mechanisms with deployable diagnostic assays.

Using bibliometric methods, Ding et al. comprehensively appraised exosome research in ischemic stroke, focusing on endogenous and therapeutic exosomes, engineered cargo, and delivery across the BBB. This data-driven landscape provides valuable references and resources to guide further exploration of exosome-based diagnostics and therapeutics.

This Research Topic delineates some research hotspots of genetics in cerebral circulation and relevant diseases. The Research Topic spans bibliometric analysis and methodological reviews (Liang et al.; Ding et al.), low-cost hematologic ratios for risk stratification and prognosis (Huang et al.; Li et al.; Ye et al.; Wang J. et al.), endocrine and rehabilitation-related markers (Mitra et al.; Xu H. et al.), genetic and molecular biomarkers (Wang J.-W. et al.; Wang and Liu; Liang et al.; Ding et al.), and perfusion-based imaging phenotypes linked to early clinical outcomes (Xu X. et al.). The cell-based and natural-product interventions establish a foundation for mechanism-guided therapies (Wang L.-P. et al.; Yu et al.). These contributions also advance clinical decision support by integrating inexpensive hematologic indexes with imaging and transcriptomic information, aiming to enhance diagnostic precision for acute management, etiologic classification, prognostic stratification, and rehabilitation follow-up.

**Figure 1:**
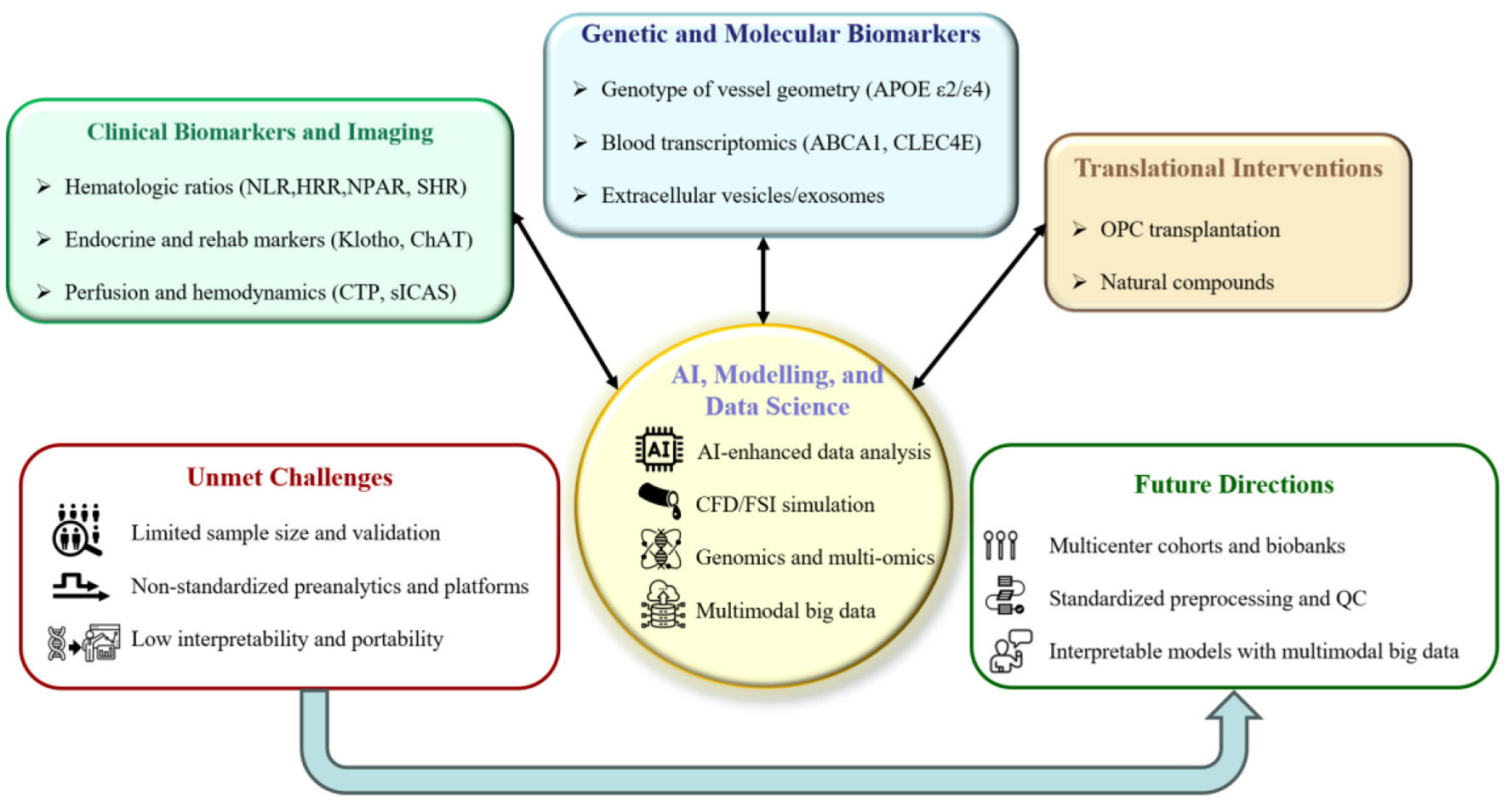
Overview of this Research Topic. The top colored panels summarize the Research Topics represented by the selected contributions: (i) clinical biomarkers and imaging; (ii) genetic and molecular biomarkers; and (iii) translational interventions. The central hub depicts AI, modeling, and data science, which are also prioritized as future directions. The bottom outline panels synthesize the limitations of the current state of the art and the corresponding priorities for future research.

Not with standing this progress, several challenges remain: insufficient availability of high-quality specimens and multicenter external validation (Mitra et al.; Huang et al.; Li et al.; Ye et al.; Wang J. et al.; Xu H. et al.; Wang J.-W. et al.; Xu X. et al.), lack of standardization in pre-analytical workflows and analytical platforms (Wang and Liu; Liang et al.; Ding et al.), limited interpretability and portability of multimodal models (Wang and Liu; Ding et al.), constraints in clinical integration and turnaround time (Liang et al.; Ding et al.), incomplete translational and regulatory pathways (Wang L.-P. et al.; Yu et al.), and ethics concerns regarding equity and accessibility (Ye et al.; Xu H. et al.). Figure 1 provides an overview of the selected contributions and maps current limitations and future research directions. Future research priorities include establishing multicenter prospective cohorts and biobanks, refining standardized preprocessing and quality-control frameworks, and developing computational models based on multimodal big data. Current efforts could be coupled with advanced computational modeling—such as computational fluid dynamics (CFD), fluid–structure interaction (FSI), and multiphysics simulation—to reconstruct cerebral and cerebrospinal fluid dynamics (1–4). Genomics and multi-omics can delineate risk loci and pathways for pathological analysis (5–8). Furthermore, interpretable artificial intelligence (AI) approaches that integrate neuroimaging, biochemical, genetic, and hemodynamic features, along with large-scale multimodal clinical data, can improve diagnostic accuracy and prognostic performance (9–13). In parallel, mechanism-anchored early-phase translation should be accelerated to support the development and evaluation of targeted interventions. Taken together, these computational and data-driven approaches will enable mechanistic elucidation, early diagnosis, and the optimization of interventions toward individualized therapy and precise medicine.
