# Supplementary material for: Editorial: Genetic insights and diagnostic innovations in cerebrovascular and cerebrospinal fluid disorders
Source: Front Neurol. 2025 Oct 21;16:1691759. doi: 10.3389/fneur.2025.1691759 (PMC12583933; doi:10.3389/fneur.2025.1691759)
Supplement: Supplementary file 1 [file Table_1.doc]

Supplementary Table 1. Summar of included studies.

| **Theme** | **Authors & DOI** | **Study Title** | **Key Findings** |
| --- | --- | --- | --- |
| Functional recovery biomarkers | Mitra et al. (2025)^[1]^ doi:10.3389/fneur.2025.1568401 | Plasma cholinergic markers are associated with post-stroke walking recovery-revisiting the STROKEWALK study | SMS-guided exercise improves 6MWT and limits BDNF decline, while cholinergic indices track walking gains and may serve as rehabilitation biomarkers. |
| Hematologic marker HRR in older AIS | Huang et al. (2025)^[2]^ doi:10.3389/fneur.2025.1534564 | Association between the hemoglobin-to-red cell distribution width ratio and three-month unfavorable outcome in older acute ischemic stroke patients: a prospective study | A lower HRR was correlated with a higher risk for adverse outcome in older AIS patients. |
| Hematologic ratios in AIS prognosis | Li et al. (2025)^[3]^ doi:10.3389/fneur.2025.1542889 | Values of lymphocyte-related ratios in predicting the clinical outcome of acute ischemic stroke patients receiving intravenous thrombolysis based on different etiologies | NLR predicts post thrombolysis outcomes across subtypes, especially in atherosclerotic stroke, supporting its use for risk stratification. |
| Inflammatory index NPAR and stroke | Ye et al. (2025)^[4]^ doi:10.3389/fneur.2025.1520298 | Cross-sectional study on the association between neutrophil-percentage-to-albumin ratio (NPAR) and prevalence of stroke among US adults: NHANES 1999-2018 | Higher NPAR is independently associated with increased stroke prevalence. |
| Metabolic stress in acute brain injury | Wang et al. (2025)^[5]^ doi:10.3389/fneur.2025.1552462 | The stress hyperglycemia ratio as a predictor of short- and long-term mortality in patients with acute brain injury: a retrospective cohort study | SHR independently predicts short- and long-term mortality in ABI and, with GCS and ventilation status, improves clinical risk stratification. |
| Endocrine biomarker (Klotho) | Xu et al. (2025)^[6]^  doi:10.3389/fneur.2025.1573027 | The association between serum klotho protein and stroke: a cross-sectional study from NHANES 2007-2016 | Serum klotho showed an independent inverse association with stroke prevalence, consistent across most subgroups. |
| Vascular genetics and geometry | Wang et al. (2025)^[7]^ doi:10.3389/fneur.2025.1498613 | Association between Apolipoprotein E gene polymorphism and the tortuosity of extracranial carotid artery | ApoE gene polymorphism are associated with the tortuosity of ECA,ε2 tended toward greater tortuosity, whereas ε4 appeared protective. |
| Shared genomic signatures | Wang et al. (2025)^[8]^ doi:10.3389/fneur.2025.1567902 | Exploration of the shared gene signatures and molecular mechanisms between cardioembolic stroke and ischemic stroke | ABCA1, CLEC4E, and IRS2 were identified as potential key biomarkers and therapeutic targets for CS and IS. |
| Imaging and perfusion in ICAS | Xu et al. (2025)^[9]^ doi:10.3389/fneur.2025.1551364 | Associations of cerebral perfusion with infarct patterns and early neurological outcomes in symptomatic intracranial atherosclerotic stenosis | Perfusion compromise aligned with border-zone and territorial infarcts, and greater penumbra-core mismatch (Tmax > 4 s) predicted early outcomes. |
| Cellular therapy in stroke | Wang et al. (2025)^[10]^ doi:10.3389/fneur.2025.1583982 | Oligodendrocyte precursor cell transplantation attenuates inflammation after ischemic stroke in mice | Oligodendrocyte precursor cell transplantation reduces neuroinflammation and preserves the blood-brain barrier in AIS, supporting its therapeutic potential. |
| Natural compounds and microglia | Yu et al. (2025)^[11]^ doi:10.3389/fcell.2025.1580479 | Targeting microglia polarization with Chinese herb-derived natural compounds for neuroprotection in ischemic stroke | Natural compounds that dampen microglia-mediated inflammation while promoting neuroprotective polarization represent a promising therapeutic strategy for IS. |
| Blood biomarkers review | Liang et al. (2025)^[12]^ doi:10.3389/fneur.2025.1488726 | Advances in the detection of biomarkers for ischemic stroke | Surveyed blood biomarkers across diverse biological pathways and underscored challenges and clinical applicability. |
| Bibliometric and research trends | Ding et al. (2025)^[13]^ doi:10.3389/fneur.2025.1595379 | Knowledge mapping of exosomes in ischemic stroke: a bibliometric analysis | The research hotpots revealed in knowledge mapping include the role of endogenous exosomes in initiating and progressing ischemic stroke, as well as the potential therapeutic applications of exogenous exosomes. |

Notes: SMS: short-message-service; 6MWT: six-minute walk test; BDNF: brain-derived neurotrophic factor; HRR: hemoglobin-to-red cell distribution width ratio; AIS: acute ischemic stroke; NLR: neutrophil-to-lymphocyte ratio; NPAR: neutrophil-percentage-to-albumin ratio; ABI: acute brain injury; GCS: Glasgow Coma Scale; ApoE: Apolipoprotein E; ECA: extracranial carotid artery; CS: cardioembolic stroke; ICAS: intracranial atherosclerotic stenosis; IS: ischemic stroke.
